# Supplementary figures and images for: COREMIC: a web-tool to search for a niche associated CORE MICrobiome
Source: PeerJ. 2018 Feb 15;6:e4395. doi: 10.7717/peerj.4395 (PMC5816963; doi:10.7717/peerj.4395)

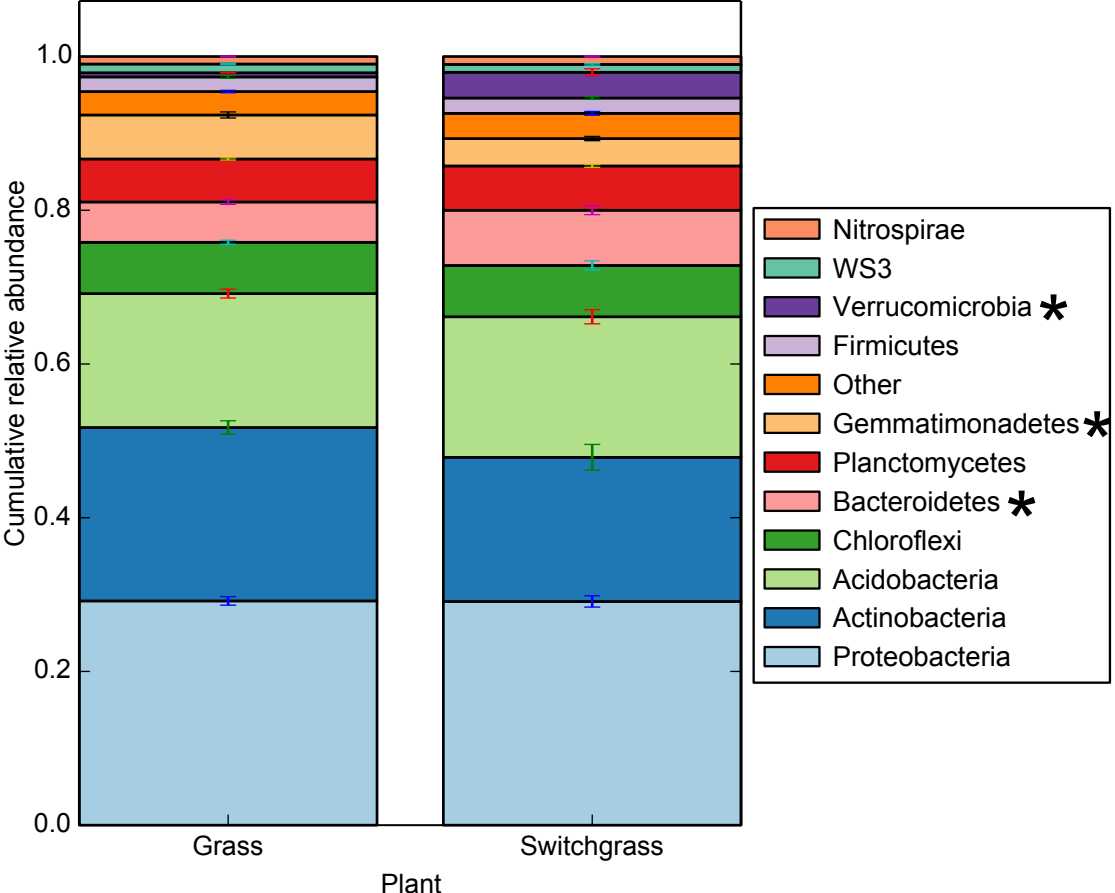

Supplement: Figure S1 — The taxa and the labels are arranged as per total relative abundance across all samples, with the most abundant phyla at the bottom and the least abundant phyla at the top of the y-axis. Mann Whitney test was used to identify phyla with significantly different (p value <0.05) relative abundance. [file peerj-06-4395-s001.pdf]

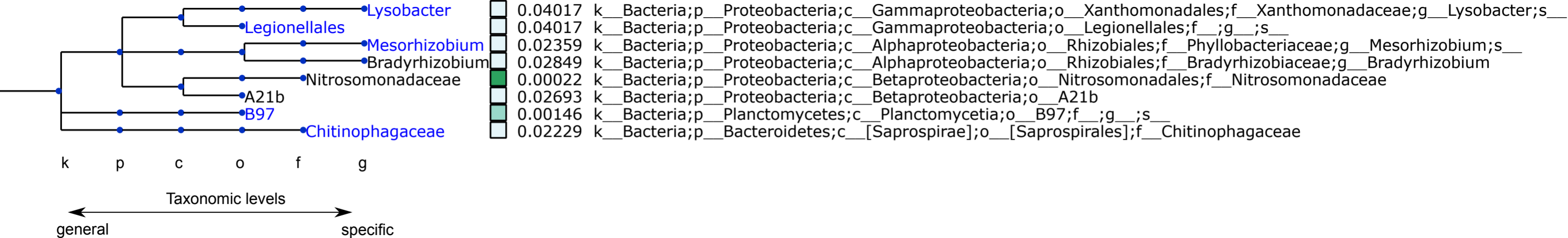

Supplement: Figure S3 — Phylogenetic tree showing relationships between core OTUs (90% threshold and q-value <0.05) identified from switchgrass (blue colored leaf label) and non-switchgrass (black colored leaf label) samples. Each level (dot) corresponds to one level of taxonomic classification (kingdom, phylum, class, order, etc.) and generated based on the taxonomy string given in the OTU abundance file. The q values and green color intensity indicate significance of the OTU being a core microbiome for switchgrass (blue colored leaf label) and non-switchgrass (black colored leaf label) samples. [file peerj-06-4395-s003.pdf]
