# Supplementary material for: COREMIC: a web-tool to search for a niche associated CORE MICrobiome
Source: PeerJ. 2018 Feb 15;6:e4395. doi: 10.7717/peerj.4395 (PMC5816963; doi:10.7717/peerj.4395)
Supplement: Figure S2 — The bar plot compares the relative abundance of switchgrass (red colored) core OTUs (90% threshold and q-value <0.05) and non-switchgrass (yellow colored) samples. The OTUs are arranged on the X-axis as per decreasing abundance in switchgrass samples, and the OTU ID (from the input table) with the taxonomic assignment is provided in the legend. [file peerj-06-4395-s002.pdf]

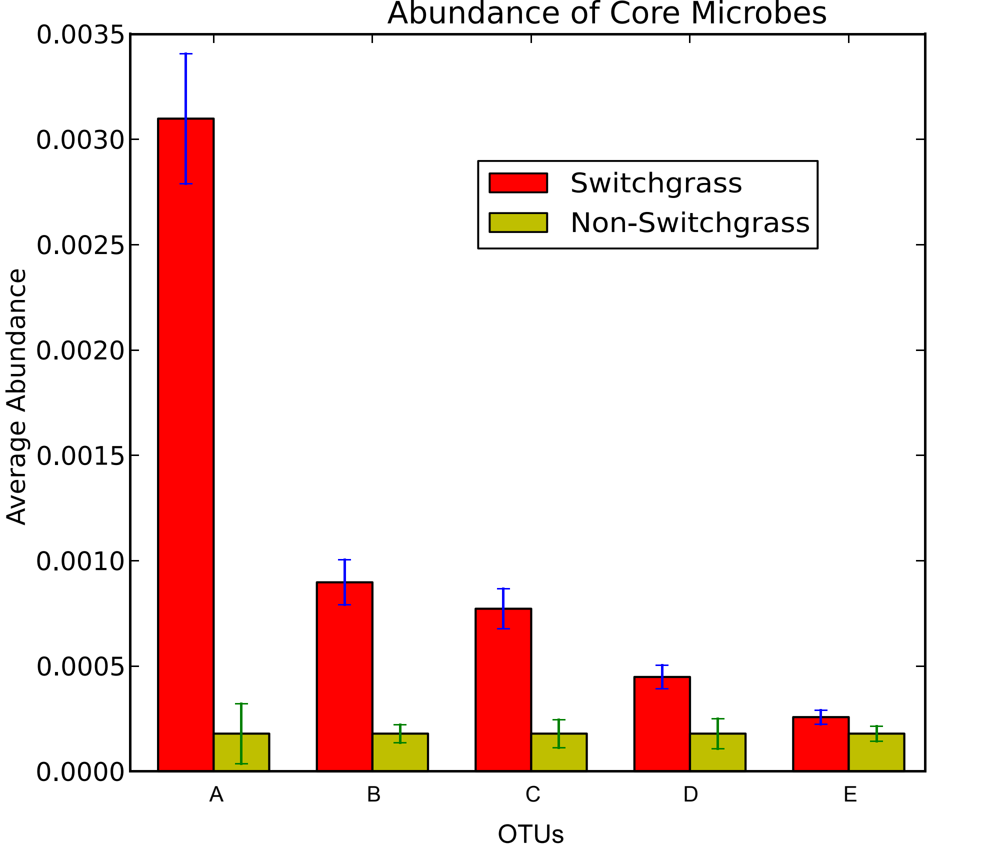

A: OTU 629	p_Proteobacteria;c_Gammaproteobacteria;o_Xanthomonadales;f_Xanthomonadaceae;g_Lysobacter;s_
B: OTU 470	p_Proteobacteria;c_Alphaproteobacteria;o_Rhizobiales;f_Phyllobacteriaceae;g_Mesorhizobium;s_
C: OTU 606	p_Proteobacteria;c_Gammaproteobacteria;o_Legionellales;f_;g_;s_
D: OTU 199	p_Bacteroidetes;c_[Saprospirae];o_[Saprospirales];f_Chitinophagaceae
E: OTU 417	p_Planctomycetes;c_Planctomycetia;o_B97;f_;g_;s_
