# Supplementary material for: COREMIC: a web-tool to search for a niche associated CORE MICrobiome
Source: PeerJ. 2018 Feb 15;6:e4395. doi: 10.7717/peerj.4395 (PMC5816963; doi:10.7717/peerj.4395)
Supplement: Table S1 — The run times (in seconds) for different sized inputs with a 678 OTUs (rows) and 59 samples (columns) dataset using default settings for COREMIC. [file peerj-06-4395-s004.docx]

**Table S1: Processing times for COREMIC.**

| **Rows = 678*numb** | **Cols = 59*numb** | **Trial 1** | **Trial 2** | **Trial 3** | **Trial 4** | **Trial 5** | **Trial 6** | **Mean** | **Std. Error** |
| --- | --- | --- | --- | --- | --- | --- | --- | --- | --- |
| 1 | 1 | 13.102 | 12.017 | 12.015 | 12.314 | 11.924 | 11.603 | 12.163 | 0.210 |
| 2 | 1 | 28.426 | 26.511 | 27.832 | 28.623 | 25.742 | 30.245 | 27.896 | 0.655 |
| 10 | 1 | 37.913 | 84.115 | 41.965 | 70.986 | 43.540 | 46.456 | 54.163 | 7.671 |
| 1 | 2 | 12.924 | 13.924 | 12.914 | 14.639 | 16.016 | 17.961 | 14.730 | 0.802 |
| 1 | 10 | 30.127 | 41.331 | 24.405 | 32.020 | 34.582 | 48.253 | 35.120 | 3.467 |
| 2 | 2 | 29.118 | 29.512 | 29.586 | 34.621 | 36.447 | 35.057 | 32.390 | 1.359 |

The run times (in seconds) for different sized inputs with a 678 OTUs (rows) and 59 samples (columns) dataset using default settings for COREMIC.
